# Optimization of Regional Water-Energy-Food Systems Based on Interval Number Multi-Objective Programming: A Case Study of Ordos, China

**DOI:** 10.3390/ijerph17207508

**Published:** 2020-10-15

**Authors:** Junfei Chen, Ziyue Zhou, Lin Chen, Tonghui Ding

**Affiliations:** 1 Business School, Hohai University, Nanjing 211100, China; zhouziyue@hhu.edu.cn (Z.Z.); 18252150037@163.com (L.C.); dingtonghui@hhu.edu.cn (T.D.); 2 Yangtze Institute for Conservation and Development, Hohai University, Nanjing 210098, China; 3 Research Institute of Jiangsu Yangtze Conservation and High-Quality Development, Nanjing 210098, China

**Keywords:** water-energy-food system, interval number multi-objective programming, optimization research, Ordos

## Abstract

Water, energy, and food are the most important basic resources for economic and social development. In the context of global population growth, environmental degradation, and resource shortages, the interrelationship between the water, energy, and food has become increasingly important. In this paper, the city of Ordos in China was selected as a study area. Firstly, this paper sorted out relevant research literature and fully understood the concept of water-energy-food (WEF) nexus. Then, an optimization model of WEF system was constructed based on interval number multi-objective programming, which took the comprehensive coordination deviation degree of the WEF system security and carbon dioxide emission minimization as the target. At the same time, the optimization model was constructed with full consideration of constraints such as economic benefit, water resource consumption, energy production, food production and environmental pollution emission. The results showed that the production of coal, thermal power, hydropower, wind power, and food all show an upward trend. Among them, the production of hydropower has the largest change, and the food production has the smallest change. In terms of water resource utilization, food production has the largest allocation of water resources accounting for nearly 80%, followed by coal production, thermal power generation and hydropower generation. The smallest allocation is natural gas. In particular, the allocation of water for coal production and hydroelectric power generation has increased significantly. Finally, the policy recommendations were put forward to promote the sustainable development of WEF system in Ordos. The optimization research on the WEF system can help to ensure the WEF system security in Ordos and promote the sustainable development of WEF system, which also can provide reference for other regions.

## 1. Introduction

**Figure 1:**
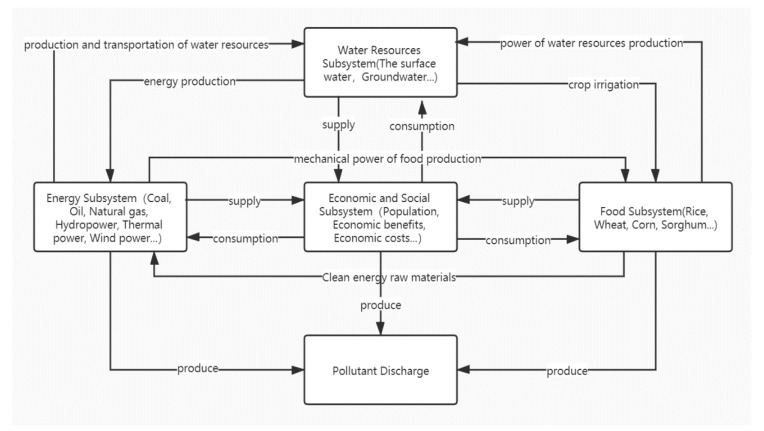
Diagram of Subsystem Relationships in WEF.

Water, energy and food, as important resources to meet people’s basic living needs, play a vital role in social and economic development [1,2]. Since the beginning of the 21st century, water crises, growing demand for energy and food shortages have affected countries around the world to varying degrees [3]. At the beginning of 2011, the World Economic Forum released the “Global Risk Report“, in which the “Water-Energy-Food“ risk group was regarded as one of the three key risk groups [4]. In the same year, the Bonn Conference used “nexus” to summarize the relationship between water, energy and food for the first time [5]. There has been formed a sensitive and fragile relationship between water, energy and food [6]. In the (water-energy-food) WEF system, the access, transport and treatment of water resources need the support of energy. Water resources are also an essential element in the process of energy processing, mining and cooling. In the process of food production, processing and irrigation, a lot of water resources are needed. Meanwhile, the production, storage and processing of food also require the supply of energy, and food can be transformed into biomass energy as a form of energy. In addition, environmental pollution is also needed to be considered into the WEF system, which mainly includes the discharge of pollutants in the process of energy and food production [7]. The relationship between the various subsystems in the WEF system is shown in Figure 1.

At present, academia has conducted a lot of research on the WEF from different angles and different methods, which focused on explaining the association relationship between water, energy and food and the quantification of the association relationship. On the one hand, it is usually to delineate the boundaries of the system and explain the relationships based on qualitative methods [2,8,9,10]. Amorim [11] defined the relationship between water, energy and food, and analyzed the impact of global risks on the relationship between water, energy and food. Hoff [12] elaborated on the definition of the WEF nexus and believed that the production and consumption of the three resources need to be coordinated to enhance. Nhamo et al. [13] believed that the conceptual framework of WEF nexus has played an important role in multi-sector coordination, management and cooperation. Both Howells et al., and Chen et al., studied the influence of factors in the WEF system through case studies [14,15]. On the other hand, due to the difficulty of obtaining simulation data and the methodological obstacles of the quantification method of the association relationship, there are few existing quantitative researches. Xu et al. [16] assessed the coupling and coordination degrees of the core WEF nexus and identified key factors that affect sustainable development. Chen et al. [17] selected Inner Mongolia as a research case, in which a super-efficiency slack based measure model and the Malmquist–Luenberger index was adopted. Both Sahin and Li [18,19] used the method of system dynamics simulation to try to quantitatively study the WEF-Nexus system.

At present, studies on optimization of WEF nexus system are quite rare. Most scholars have considered the optimization of a single resource, rather than the optimization of WEF as a whole. In terms of single resource or two resources optimization, Javadinejad et al., used different methods and models to optimize water resources [20,21,22]. Ju and Su et al., all proposed a multi-objective model to optimize energy [23,24]. Guo and Cai respectively build models to optimize the food planting structure to better adapt to the changes in demand [25,26]. Cao et al. [27] optimized the structure of planting industry in arid areas under the constraints of water resources. Zhen et al. [28] constrained a hybrid interval two-stage fuzzy credibility programming model for supporting energy-water nexus system management. Chen et al. [29] proposed a dual risk aversion optimization method—an energy water nexus system model, which can generate robust optimization solutions. In terms of the comprehensive optimization of water, energy and food, Zhang [30] optimized water and energy utilization and food production based on the comprehensive evaluation method. Karan [31] established a system and proposed a mathematical model for forecasting demand and production containing water, energy and food. Fan [32] conducted an integrated evaluation of the WEF nexus for two areas with different levels of urbanization using empirical multiple linear regression in a simultaneous equation model. Li [33] established an optimization model with the goal of maximizing economic benefits and minimal environmental impact. Peng [34] optimized the relationship between water, energy and food basin by introducing the principle of synergetic According to the literature review, it can be observed that there is a lack of studies on the overall optimization of the region WEF. At the same time, less attention is paid to the uncertainty of the operation of the WEF system and the discharge of environmental pollutants. This paper considered WEF as a comprehensive system, while incorporating economic benefits and ecological environment.

Multi-objective programming involves multiple theories and disciplines, which is widely used in many fields such as social economic production, engineering design and environmental protection [35]. The idea of multi-objective programming originated from the utility theory in economic research in 1776. In 1896, Pareto [36] proposed the concept of multi-objective programming for the first time. In 1947, Vonneuman and Morgenstern [37] described the problem of multi-objective programming in their game theory work. Since then, the idea of multi-objective programming has gradually attracted widespread attention. So far, more and more scholars have stepped into the field of multi-objective optimization research and have achieved many gratifying achievements [38,39,40,41]. Therefore, this paper used multi-objective functions to optimize the WEF system. Interval number programming expresses the uncertainty of parameters in the form of interval numbers [42]. In the optimization of interval number programming, the possible variation range of any uncertain parameter is represented by an interval, which is only the upper and lower limits of the parameter need to be known, and the precise probability distribution or fuzzy membership function is not needed [43]. The interval range covers a variety of advantages in terms of uncertainty and complexity [44]. Therefore, this paper used interval numbers to transform the coupling relationship in the WEF system and some uncertain factors into measurable factors.

At present, studies on the optimization of WEF system are quite rare, which are not enough to support the optimization research of the WEF system security. It is the keys to constructing the optimization model of WEF system that can characterize uncertain factors and conducting research on WEF system security optimization to promote the sustainable development of WEF systems. Therefore, this paper took Ordos, an arid and water-deficient area of Inner Mongolia, as an example and conducted a study on the optimization of the WEF system security in Ordos. And multi-objective programming model and interval number programming model were combined in this paper. This research can provide a theoretical framework and technical support for the sustainable development of the WEF system in Ordos. The paper is organized as follows: Section 2 introduces the study area. Section 3 describes the methods and data. The main results and discussion are presented in Section 4. Section 5 gives the conclusions and suggestions.

## 2. Study Area and Data Sources

**Figure 2:**
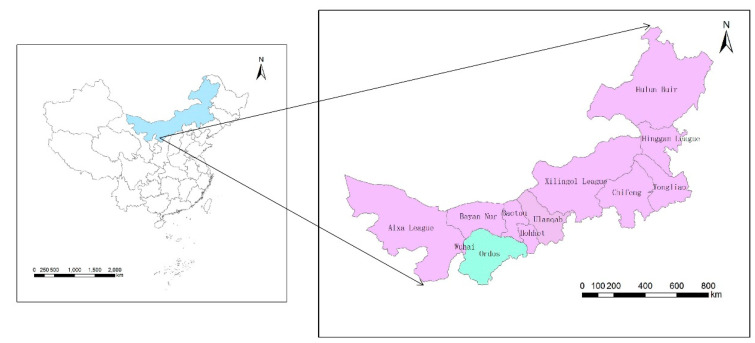
The location of Ordos in Inner Mongolia, China.

Ordos is located in the southwestern part of Inner Mongolia in China, covering an area of 86,572 km^2^ (Figure 2). In terms of economic development, the economy of Ordos has developed rapidly in recent years. In 2017, the city’s regional GDP reached 357,981,000,000 yuan, ranking first in the Inner Mongolia Autonomous Region. In terms of the development and utilization of water resources, Ordos had a total of 2,142,000,000 billion m^3^ of water resources in 2017, accounting for only 7% of the total water resources in Inner Mongolia Autonomous Region. The per capita amount of water resources was 1000 m^3^, which was lower than the regional and national average. In terms of energy production, Ordos has more than 50 types of mineral resources. The proven coal and natural gas reserve account for 1/6 and 1/3 of the country [45]. Ordos, as a demonstration area in China’s “13th Five-Year” coal deep processing plan, carries high-intensity energy development and requires a lot of water resources. In terms of food production, the food production in Ordos is dominated by wheat, potatoes, corn, sorghum and soybeans. In recent years, the total food production has risen steadily. The shortage of water resources in Ordos has always restricted its food production, which has caused some problems in Ordos’ food production. The large-scale use of pesticides and fertilizers in food production has severely affected the ecological environment of land and water resources. Therefore, it can be seen that Ordos is a typical region for studies on the WEF nexus.

However, the rapid development of the energy industry will put pressure on the provision of water resources in Ordos. Besides, food and energy departments are the main departments of water consumption in Ordos. How to reasonably allocate energy and food production and the distribution of water resources in energy and food will become the top priority of the economic and social development of Ordos [46]. In addition, the vegetation coverage of Ordos has improved to some extent, but ecological problems such as soil erosion, desertification, soil salinization, and wetland shrinkage are still outstanding in recent years. Therefore, how to improve the ecological environment in the implementation of economic development is also an urgent problem that Ordos needs to solve.

The data resources of this study are mainly the parameters in the model. The data sources include the Statistical Yearbook of Ordos City, National Economic and Social Development Statistical Bulletin of Ordos City, Environmental Bulletin of Inner Mongolia, 13th Five-Year Plan, Water Resources Bulletin of Inner Mongolia and relevant plans and documents.

## 3. Methodology

Regarding the application of interval number linear programming models in reality, there may not be only one objective function. In this case, the interval number multi-objective programming model needs to be used. Interval number multi-objective programming model, which is the combination of interval number programming model and multi-objective programming model. The interval number multi-objective programming model can characterize the uncertainty in the actual problem, and then solve the model to obtain a set of decision variable intervals. When guiding decision-making based on the solved decision variable intervals, specific decision-making schemes can be determined in combination with actual conditions and experiences [47]. The decision-making scheme obtained by this method is more feasible and operable [48]. At the same time, it is closer to reality than the interval number programming model and can better solve practical problems. Due to the uncertainty of its index factors and the diversity of goals in WEF system, interval number multi-objective programming has good applicability to the study of WEF system security. In order to carry out the optimization research of the WEF system security in Ordos, this paper constructed an interval number multi-objective planning model, which took minimum WEF system security comprehensive coordination deviation and minimum carbon dioxide emissions as the goal, energy and food production and the allocation of water resources in energy and food production as decision variables. The optimized production value of energy and food production and the optimized distribution value of water resources can be obtained in Ordos by solving the model.

### 3.1. Decision Variables

Assuming *X_i_* = (*x*_1_, *x*_2_, …, *x*_11_), where (*i* = 1, 2, …, 5) represents the annual production of the *s*-th energy. *x_i_*(*i* = 1, 2, …, 5) represents coal mining, natural gas mining, thermal power generation, hydroelectric power generation, wind power generation. *x_i_* (*i* = 6) represents the annual food production. The food range is taken from the general categories of food range determined in the Ordos Statistical Yearbook. *x_i_* (*i* = 7, …, 10) represents the annual allocation of water resources for coal mining, natural gas mining, thermal power generation, and hydropower generation. *x_i_* (*i* = 11) is the annual allocation of water resources for food production.

### 3.2. Objective Function

WEF system security is to group water security, energy security and food security as a whole from the perspective of the system, which maximize the overall benefit of water-energy-food. The WEF system security in this paper means that the regional water resources, energy and food system can maximize the overall benefit of the complex system through the coupling coordination among the three on the premise of ensuring the sustainable development of each subsystem, and minimize the negative impact on the environment. This paper takes the minimum comprehensive coordination deviation of WEF system security as the overall goal. The deviation degree of WEF system includes the degrees of deviation of the energy subsystem, food subsystem and water resource subsystem, respectively. Degree of deviation refers to the deviation between the planned quantity and the actual quantity. The smaller the degree of deviation is, the higher the coordination of the WEF system security is. In addition, it is necessary to emphasize the treatment of environmental pollutant emissions during economic development. The pollutant elements in the WEF system mainly use several types of pollutants with high pollution emissions as environmental pollution elements, including wastewater, chemical oxygen demand (COD), carbon dioxide, nitrogen oxides, smoke (dust) emissions, etc. The country attaches great importance to low-carbon economy. However, due to the production and consumption of energy, and the production of food, a large amount of carbon dioxide has been produced. Due to the current carbon dioxide emission reduction has great technical difficulties and other environmental pollutants treatment technologies are relatively mature, such as sulfur dioxide. Therefore, carbon dioxide is selected as the environmental pollution emission, and taking the minimum carbon dioxide emission as the environmental goal. Other pollution is not considered in this paper. The specific equation is as follows:

(1) $$\min F_{1}=\sum_{i=1}^{11}w_{i}\times \left|\frac{S_{i}-x_{i}}{S_{i}}\right|$$

(2) $$\min F_{2}=\sum_{i=1}^{6}K_{i}\cdot x_{i}$$

Here, *S_i_*(*i* = 1, 2,…, 5) represents the planned production of coal, natural gas, thermal power, hydropower, and wind power, respectively. *S*_6_ is food planning production. *S*_i_ (*i* = 7, 8,…, 11) denotes the amount of water resources available for coal, natural gas, thermal power generation, hydropower generation, and food production. *w_i_* (*i* = 1, 2, …, 11) is the contribution weight of energy, food and water resources to the WEF comprehensive coordination goal, which are determined by the analytic hierarchy process method (AHP). *K_i_*(*i* = 1, 2, …, 6) represents the carbon emission coefficient of energy and food production. *K_i_* (*i* = 1, 2, …, 5) represents the coal carbon emission coefficient, natural gas carbon emission coefficient, thermal power generation carbon emission coefficient, hydropower generation carbon emission coefficient and wind power generation carbon emission coefficient. *K*_6_ represents the food production carbon emission coefficient. The carbon emission factor refers to the amount of carbon emissions per unit energy produced during the use of each energy.

### 3.3. Restrictions

After the objective function is determined, in order to ensure the orderly operation of the WEF system, various constraints are determined, which include economic benefit constraints, water resource consumption constraints, energy production constraints, food production constraints, environmental pollution emissions constraints, and decision variables.

#### 3.3.1. Economic Benefit Constraints

Economic constraints mainly refer to economic cost constraints in various energy production and food production processes. The energy production cost must be less than the maximum energy production cost. Food production cost must be less than the maximum food production cost:

(3) $$\sum_{i=1}^{5}C_{i}\times x_{i}\leq C^{e}\;i=1,2,\cdots ,5$$

(4) $$C_{6}\times x_{6}\leq C^{f}$$

Here, *C_i_* represents the unit energy production cost of the *i*-th energy. *i* (*i* = 1, 2, …, 5) represents unit coal mining cost, unit natural gas mining cost, unit thermal power generation cost, unit hydroelectric power generation cost, unit wind power generation cost. s represents the maximum energy production cost. *C^e^* represents the maximum energy production cost. *C*_6_ and *C^f^* represent the unit food production and the maximum food production cost.

#### 3.3.2. The Supply and Demand of Water Resource Constraints

Water resources are the key elements in energy production and food production. In order to ensure the security of WEF system and avoid the waste of water resources in energy and food production, the model constructs water resource utilization constraints. The main constraints of water resources utilization are as follows. Firstly, the amount of water used for energy and food production must not exceed the available amount of water resources (Equation (5)). Secondly, the amount of water consumed for energy and food production needs to be greater than that for energy and food production in order to meet the stable development of energy and food (Equations (6) and (7)):

(5) $$\sum_{i=1}^{6}H_{i}\cdot x_{i}\leq 83\%\cdot TW\;i=1,2,\cdots ,7$$

(6) $$\sum_{i=7}^{10}x_{i}\geq WD^{e}$$

(7) $$x_{11}\geq WD^{f}$$

where *H_i_*(*i* = 1, 2,…, 5) 6 represents the coal utilization water resources utilization coefficient, natural gas exploitation water resources utilization coefficient, thermal power generation water resources utilization coefficient, hydropower generation water resources utilization coefficient, wind power generation water resources utilization coefficient, respectively. *H_i_* (*i* = 6) denotes the utilization coefficient of water resources for food production. *TW* is the availability of water resources. Take the proportion of farmland and industrial water in total water consumption as the proportion of food production and energy production water in total water consumption, which is about 83%. *WD^e^* represents the water demand for energy production. *WD^f^* is the water requirement for food production.

#### 3.3.3. Energy Production Constraints

The main constraint for energy production is that the total regional energy production must not exceed resource reserves and production capacity. And the regional energy production meets the energy production requirements and energy self-sufficiency requirements of the 13th Five-Year Plan:

(8) $$PC_{\min}\leq x_{1}\leq PC_{\max}$$

(9) $$x_{2}\geq PG$$

(10) $$x_{3}\geq PT$$

(11) $$\frac{\sum_{i=1}^{5}x_{i}}{EC}\geq ESR$$

(12) $$\sum_{i=3}^{5}x_{i}\geq PP$$

Here, *PC*_min_ represents the minimum coal production requirement, *PC*_max_ represents the coal production capacity, *PG* represents the minimum natural gas production requirement, *PT* is the minimum thermal power generation production requirement, *EC* represents the energy consumption and *ESR* represents the energy self-sufficiency rate requirement, *PP* represents the power production requirement.

#### 3.3.4. Food Production Constraints

The main constraints for food production are as follows: (1) The regional food production reaches the minimum food production requirement. (2) The regional food planting area is larger than the food production guarantee area. (3) The per capita minimum food production limit. (4) The regional food self-sufficiency rate reaches a certain level. (5) The amount of chemical fertilizer used should be less than the maximum value of the amount of chemical fertilizer. (6) The amount of energy used in food production is less than or equal to the amount of energy distributed in food production. (7) The amount of water distribution of per unit irrigation area should reach a certain guarantee:

(13) $$x_{6}\geq PF_{\min}$$

(14) $$\frac{x_{6}}{PFA}\geq BFA$$

where, *PF*_min_ represents the minimum food production. *PFA* denotes the food production per unit area and *BFA* is the food production guarantee area:

(15) $$\frac{x_{6}}{TP}\geq RSF$$

(16) $$\frac{x_{6}}{FC}\geq FSR$$

(17) $$PFA\times x_{6}\leq TFA$$

Here, *TP* represents the total food population. *RSF* represents the lowest per capita food production. *FC* denotes the food consumption. *FSR* is the food self-sufficiency rate requirement. *PFA* represents the amount of fertilizer application required per unit food production and *TFA* represents the maximum value of fertilizer application.

#### 3.3.5. Environmental Emission Constraints

Referring to Zhang‘s research on WEF for environmental emission constraints, the model considers carbon dioxide as the main pollutant generated during the operation of energy and food subsystems in the WEF system [49]:

(18) $$\sum_{i=1}^{5}K_{i}\cdot x_{i}\leq PE_{CO_{2}}^{e}\;i=1,2,\cdots ,5$$

(19) $$K_{6}\cdot x_{6}\leq PE_{CO_{2}}^{f}$$

Here, $PE_{CO_{2}}^{e}$ and $PE_{CO_{2}}^{f}$ represent the allowable carbon dioxide emissions of the energy subsystem and the food subsystem, respectively.

#### 3.3.6. Non-Negative Constraint

All decision variables are non-negative variables:

(20) $$x_{i}\geq 0\;i=1,2,\cdots ,11$$

## 4. Results and Discussion

This paper refers to the related literature and solves the multi-objective programming problem with interval numbers through a two-step method. Firstly, the parameters should be estimated. Secondly, we have to determine the target expectation level. Finally, the target value of each target, the achievement level of each target and the value of each decision variable under each target value can be obtained by introducing the concept of satisfaction. The above solution process is realized by Lingo, which is used to solve nonlinear programming, linear and nonlinear equations, etc. and is the best choice for solving optimization models.

### 4.1. Parameter Estimation

Based on the 13th Five-Year Plan of Ordos and economic and social development indicators, this paper selected 2017 as the horizontal year and 2020 as the planned level year. Among them, the horizontal year is the base year of the plan, which is the year when your current data is collected, and all subsequent forecasts are based on the horizontal year. The planned level year is the target year of planning. According to the current status of water, energy, and food production and utilization in Ordos, the parameters of optimization model of the WEF system are determined and estimated, including social economic parameters, water resources utilization parameters, energy production parameters, food production parameters, and environmental pollution parameters.

#### 4.1.1. Determination of Social Economic Parameters

For the parameter of Ordos’ population, this paper referred to the prediction of the population of Ordos in 2020 in the 13th Five-Year Plan of Ordos. For the unit energy production cost coefficient, the price of energy can indirectly reflect the production cost of energy, so the energy price of various types of energy is used to measure the production cost of energy referring to the research of Ouyang [50]. This paper predicted the unit food production cost coefficient of Ordos referring to the study of Li [51]. The maximum production cost of energy and food is estimated based on various energy production and energy cost data and the food production data of Ordos City in 2017. The specific values after unit conversion are shown in Table S1 in Supplementary Materials.

#### 4.1.2. Determination of Water Resources Utilization Parameters

For energy production, the water resource utilization factor includes water resource utilization during energy extraction and processing. Based on the actual situation of Ordos City and the research of Hong, this paper determined the water resource utilization coefficient for energy production [52]. The water resource utilization coefficient for food production was estimated, which was according to the quota for water use and the food production index per unit of arable land area in Inner Mongolia. The maximum utilization of water resources was obtained through the 13th Five-Year Plan of Ordos. Regarding the planned utilization of water resources, we can estimate the planned utilization of water resources for energy production and the planned utilization of water resources for food production in 2020 based on the product of Ordos’ energy production water utilization coefficient and energy production. The water requirements for energy and food production are determined by the water resources required for the minimum output. The specific values after unit conversion are shown in Table S2 in Supplementary Materials.

#### 4.1.3. Determination of Energy Production Parameters

The planned energy output is obtained from the 13th Five-Year Plan for Energy of Ordos City and the website of Ordos Development and Reform Commission. The minimum coal production value and capacity have been determined, which are referred to the 13th Five-Year Plan of the Ordos Coal Industry. The minimum production of natural gas and the proportion of clean energy are according to the “Development Plan for the Ordos Clean Energy Production Base” issued by the Ordos government. According to the requirements of the 13th Five-Year Plan of Ordos, this paper takes the minimum production of thermal power and wind power. In terms of total energy consumption, we calculated the growth rate of energy consumption by 6.68% by referring to Inner Mongolia Statistical Yearbook data. We can get the total annual energy consumption of Ordos. According to the “Development Plan for Clean Energy Production Base of Ordos City” issued by the Ordos Government, power production has been obtained. Energy self-sufficiency is the ratio of energy production to consumption. The specific values after unit conversion are shown in Table S3 in Supplementary Materials.

#### 4.1.4. Determination of Food Production Parameters

The planned food production in Ordos in 2020 is based on the food production in Ordos in 2017. According to the index of food production per unit of arable land area in the Ordos Statistical Yearbook, we obtained the area of arable land required per unit of food production. Through the ratio of total chemical fertilizer use to total food output, the chemical fertilizer use per unit food output is determined. This paper estimated the maximum value of chemical fertilizer application based on the application of chemical fertilizer in Ordos food production in 2017. The minimum per capita food output is based on the baseline indicators of food security regulation of the Rural Economic Research Department of the Development Research Center of the State Council. The guaranteed area of food production refers to the area of cultivated land reserved for food production, which is obtained by referring to the amount of cultivated land and the ratio of the area of food production to the total cultivated land area. Food self-sufficiency rate refers to the proportion of food production, which determined the food self-sufficiency rate by according to the Outline of the National Medium and Long-Term Planning for Food Security. The specific values after unit conversion are shown in Table S4 in the Supplementary Materials.

#### 4.1.5. Determination of Environmental Pollution Parameters

This paper calculates the carbon dioxide emissions by multiplying the carbon emission coefficient and various energy and food production. According to the data released by the Energy Research Institute of the National Development and Reform Commission, the carbon emission coefficient was obtained. Regarding the carbon emission coefficients of food production, the carbon emissions during food production mainly come from irrigation and the use of pesticides, fertilizers, and agricultural films. With reference to the studies by Li and Tian, the carbon emission coefficient of food production is estimated [53,54]. The specific values after unit conversion are shown in Table S5 in the Supplementary Materials.

### 4.2. Target Expectation Level

The multi-objective problem can be decomposed into multiple single-objective interval number linear programming problems. Then, sub-models are constructed separately for each single-objective interval number model after decomposition. Substituting the optimal solution corresponding to each single objective into all objective functions, a series of objective function values are obtained, from which the upper and lower limits of the desired target level of each objective function can be obtained. The target expectation level is shown in Table 1.

The desired level of coordination deviation of WEF system reflects the degree of coordination in the WEF system. As seen from Table 1 the lower limit 0.02 represents the optimal level of WEF system coordination deviation. And the upper limit 0.179 represents the maximum acceptable level of WEF system coordination deviation. The upper limit and lower limit of the environmental target are equal, which indicated the desired level of the ecological environmental target is a certain value in the WEF system.

### 4.3. Determine Decision Variables

According to the upper limit and lower limit of the target expectation level, decision variables under the target expectation level can be obtained, which represent the optimized production value of energy and food and the optimized distribution value of water resources in Ordos. The details are shown in Table 2.

### 4.4. Result Analysis and Discussion

Coal, as the main energy source in Ordos, is a non-renewable energy source. Although coal resources in Ordos are rich, the production process of coal consumes abundant water. A large amount of water pollution, air pollutants, and solid waste are generated during the production process, which has a greater impact on the environment. Moreover, during the “Twelfth Five-Year Plan” period, coal production in Ordos declined steadily and coal sales also showed a downward trend, which indicated that coal production can meet the needs of daily life in Ordos. Therefore, coal production should minimize coal production and reduce coal production surplus while meeting the coal supply needed for daily production in Ordos. The optimized production of energy is determined to be [464,295,000, 464,894,100] tons standard coal finally. In order to ensure energy security in the WEF system, Ordos should maintain the production of primary energy sources and promote the structural reform of the coal supply side. With a proper balance between total coal supply and guaranteed supply, the mechanism to resolve coal overcapacity should be further implemented to solve the problem of excess coal capacity and promote the sustainable use of coal resources.

Natural gas is a clean energy source, which produces less sulfur dioxide during production and consumption than other fossil fuels and requires very little water during its production process. It should be used as an energy production method to be vigorously promoted by Ordos. Therefore, the final optimized production of Ordos natural gas is [33,000,000, 39,097,840] tons of standard coal in this paper. The production of natural gas and other clean energy should be vigorously developed to promote the transformation of energy system in Ordos from traditional energy to clean energy, which can reduce the emission of environmental pollutants in the process of energy production and utilization. The development of clean energy can change the situation of over-reliance on traditional energy, which can promote the formation of a green, low-carbon, safe and efficient, shared and win-win modern energy economic system in Ordos.

Among the three power generation methods, the thermal power generation process consumes the largest amount of water and the environmental pollution caused by thermal power generation has a greater impact. In order to achieve the sustainable development of WEF, Ordos should reduce the amount of thermal power generation. Therefore, it is finally determined that the optimized production of thermal power generation is 12,674,800 tons of standard coal. Although the production process of hydropower itself consumes almost no water, due to reservoir seepage, water surface evaporation and other reasons, the demand for water resources is higher than that of other clean energy sources. Therefore, the optimized production of hydropower is determined to be 230,860 tons of standard coal in Ordos. Wind power generation is a renewable energy source that produces less pollutant. According to the minimum coordination deviation goal and minimum pollutant emissions of WEF system, wind power generation should be vigorously developed. At the same time, considering the economic cost of wind power generation in Ordos, we finally determined that the optimized production of wind power is 94,340 tons of standard coal. In order to promote the rational production of Ordos energy and the safe development of the WEF system, the Ordos energy system should be optimized and upgraded. While reducing primary energy production, scientific and technological innovation should be encouraged to reduce the production costs of clean energy and make clean energy both environmentally friendly and economically applicable, especially wind power generation.

In terms of food production, the paper finally determined that the optimized food production of Ordos in 2020 will be [1,615,010, 1,742,160] tons. According to the analysis of food production in Ordos in recent years, it can be found that the annual food production of Ordos has remained about 1.45 million tons in recent years. Moreover, the Ordos government issued the “Notice on the Delineation of Food Production Functional Zones and Important Agricultural Production Protection Zones in Ordos City”, which delineated various food production functional areas in Ordos City and determined relevant requirements for food production. Therefore, there is room for growth in food production in Ordos. However, there are also some problems in Ordos’ food production, which has been always restricted by the shortage of water resources in Ordos. In recent years, the large-scale use of pesticides and fertilizers in food production has seriously affected the ecological environment of land and water bodies, which caused the water resources are even more in short supply. In this case, Ordos should increase food production. Firstly, it should increase the protection of arable land, stabilize the planting area of food crops, and maximize the role of land resources in increasing food production. Secondly, Food growth is inseparable from the improvement of planting technology. Ordos needs to promote green production-increasing and efficient cultivation techniques for crops. Food enterprises should be supported to strengthen the research and development of new food varieties, new technologies and new processes. Thirdly, Agricultural machinery and equipment should give full play to the role in reducing costs, reducing losses and so on, so as to improve the level of food production. Finally, farmers must pay attention to the impact of land resources on food production and improve land production capacity. Too much or too little pesticides and fertilizers are not conducive to protecting the soil and increasing food production. Therefore, we must rationally use pesticides and fertilizers to promote the sustainable development of food cultivation.

**Figure 3:**
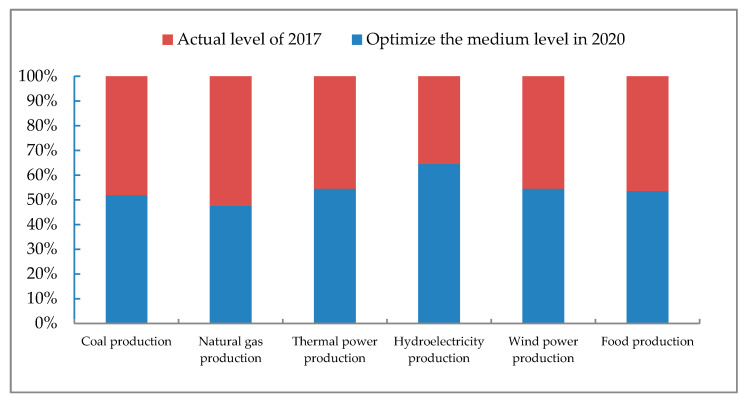
Production comparison chart between horizontal year and planned year.

According to the comparison chart of energy and food production in the horizontal year and the planned year (Figure 3), it can be seen that the production of coal, thermal power, hydropower, wind power, and food in 2020 will be greater than that of 2017, and the production of natural gas will be slightly smaller than that of 2017. Among them, there is little difference between coal production in 2017 and 2020, which shows that Ordos has played a controlling role in coal production. On the basis of basically satisfying human production and life, coal mining and production have not been carried out on a large scale. In 2017, natural gas production reached 29.93 billion m^3^, which is already higher than the level planned in this article. This is related to the continuous exploration of Ordos natural gas resources. In recent years, China has accumulatively proven 10 billion-ton oil fields, of which 9 are from the Ordos Basin. Compared with the planned production, thermal power and wind power are still slightly lower, which indicates that they should maintain steady growth. In 2017, Ordos hydropower has been below the level planned for 2020, which shows that it has realized that hydropower has excessive demand for water resources, which is inconsistent with the reality of Ordos and its water shortage. The food production in 2017 was 1.453 million tons, and there is still a certain gap between the optimized results in 2020, so Ordos needs to further stably increase food production.

**Figure 4:**
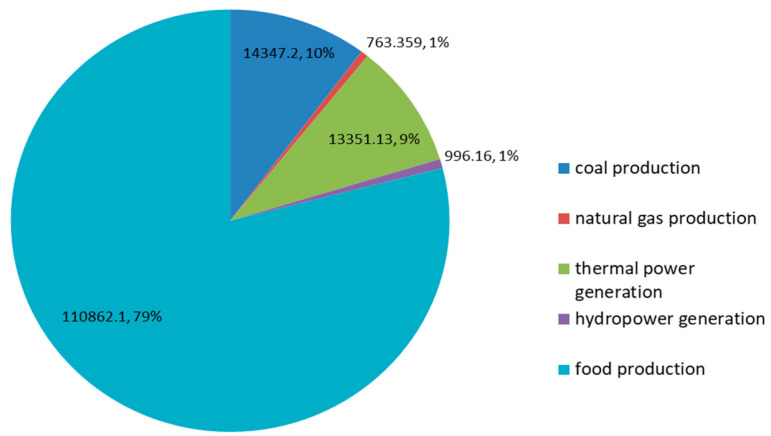
The pie chart of water resource utilization.

In terms of water resource utilization, as seen from Figure 4, food production has the largest allocation of water resources, followed by coal production, thermal power generation and hydropower generation. The smallest allocation is natural gas. Natural gas and hydroelectric power generation have a small amount of water resources distribution, while having a small impact on the ecological environment, which has a positive effect on the WEF system security in Ordos. In the distribution of water resources, the optimized amount of water used for food production accounts for about 79% of the total optimized amount of water resources. It can be seen that reducing water resources in food production plays an important role in reducing water resources. With the development of society, water resources have become the main factor restricting the economic development of Ordos. According to the Inner Mongolia Water Resources Bulletin, in recent years, the water consumption of Ordos is equal to the water supply, which shows that the water consumption of Ordos has reached the upper limit of the available water resources [55]. The rapid development of the energy industry and the food industry has put pressure on the use of water resources in Ordos City, which will exacerbate the water shortage among different industries in Ordos. Therefore, Ordos City should optimize the water resources utilization structure. Firstly, water utilization in agriculture and the energy industry is heavily restricted, which should strictly adhere to the three red lines of water resources and promote the conservation of water resources. Secondly, in the process of agricultural water use, we must deepen the control of the total amount of agricultural irrigation water and energy production. Micro-sprinkler irrigation, drip irrigation, drip irrigation and other irrigation methods should be used in local precision irrigation to improve the utilization rate of water resources. Thirdly, in the process of water use in the energy industry, the government should limit the production scale of high-water-consumption and high-polluting energy sources to reduce the pressure on water use. Next, the initial water rights distribution system and the water rights trading system should be established and improved. On this basis, the government needs to promote the trade of water rights among regions, industries and users, so as to alleviate the contradiction of water use and balance the supply and demand of water resources. Finally, we must strengthen the recycling of industrial wastewater. High water-consuming and high-polluting industries need to implement major pollutant discharge reduction and replacement work, which plays an important role in reducing the degree of water pollution and realizing water pollution control and reuse.

**Figure 5:**
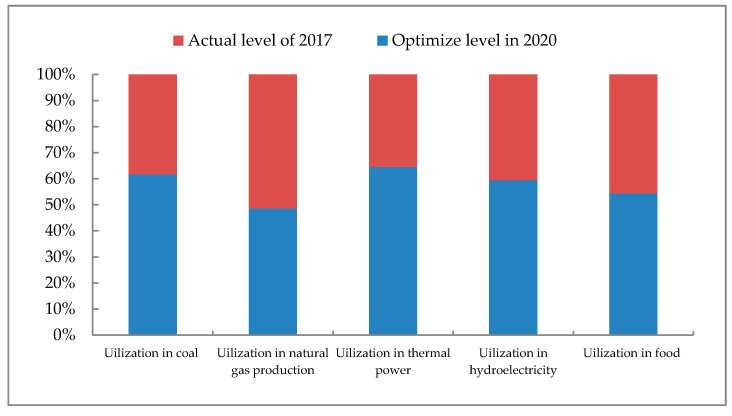
Comparison chart of water resources utilization between horizontal year and planned year.

According to the comparison chart of water resources utilization between the horizontal year and the planned year (Figure 5), we can found that except for the amount of water allocated for natural gas is higher than the planned level, the allocated amount of water for other energy and food production is lower than the planned amount for 2020, which corresponds to various types of energy and food production. Among them, the amount of water resources used for coal production was 89.424 million tons in 2017, which was only about 62% of the planned year. Which shows that the utilization efficiency of water resources has been improved in the coal production process, and the water saving effect is obvious. The water consumption of natural gas is slightly higher than the planned level, which is directly related to the high production of natural gas. However, the concept of green development should be implemented in the process of mining and processing for the sustainable development of Ordos natural gas resources and the rational use of water resources. The actual water consumption of thermal power and hydropower in 2017 is far less than the planned amount in 2020, which shows that Ordos has begun to notice the adverse effects of thermal power and water power and strive to control the use of water resources in the process of power generation. The water consumption of food production is not too different between the level year and the planned year, but Ordos still needs to further improve the efficiency of water use on the basis of increasing food production.

## 5. Conclusions

In this paper, the interval multi-objective programming model was used to optimize the safety of the Ordos WEF system, which aimed at the least deviation from system coordination and the least carbon dioxide emissions. The WEF system optimization scheme suitable for the sustainable development of Ordos was obtained. Therefore, this paper draws the main conclusions and put forward some policy suggestions to improve the WEF system security.

Firstly, it can be seen that the optimization of coal production in the planned year is not much different compared with the horizontal year (2017), which indicates that coal production should remain stable while meeting human normal production and life. At the same time, coal production has a huge impact on water consumption and environmental pollution. Therefore, the production of coal should be minimized on the basis of satisfying production and living conditions, while natural gas should be promoted as the energy production mode vigorously promoted. For the three ways of generating electricity, the planned production of thermal power and wind power is higher than the actual annual production, which shows that Ordos should steadily increase the production of thermal power and wind power. However, considering environmental pollution and lack of water resources, wind power should be encouraged first. Because of the pollution and water demand of thermal power generation, thermal power generation should be greatly reduced. Therefore, the energy system should be optimized and upgraded in Ordos. While reducing the primary energy production, new energy technologies should be reformed to further reduce the cost of new energy production

Secondly, compared with the horizontal year, the optimized food production is on the rise. Based on the analysis of Ordos’ food production in recent years, we found that there is a lot of room for growth in Ordos’ food production. Related policies and notice also provide a guarantee for the stable development of Ordos food production. However, the shortage of water resources in Ordos has always restricted food production, and the large-scale use of pesticides and fertilizers in food production has seriously affected the ecological environment of land and water bodies in recent years. Therefore, factors such as per capita food possession, food production water consumption and pollutant emissions should be considered. In addition, the area of food planting should be expanded to give full play to land resources. Improving the level of food production through the improvement of planting technology and the innovation of food enterprises is necessary. At the same time, pesticides and fertilizers are used rationally to promote the sustainable development of food cultivation.

Finally, compared with the horizontal year (2017), planned allocation of water resources for coal production, thermal power, hydropower, and food production have all improved significantly, and the amount of natural gas production and allocation is not much different, which shows that the demand for water for various energy and food production in Ordos is still increasing. Water resources are the most unfavorable factor in the development of Ordos. It can be found that the optimized amount of water used for food production accounts for about 80% of the total, which implies that there is great potential for reducing the total water use by reducing the water consumption for food. On the one hand, the water resources utilization structure in Ordos should be optimized. All industries need to strictly restrict water use and increase the efficiency of water use. On the other hand, the government should strengthen the management and control of the recycling of industrial wastewater.

In this paper, the optimization study was carried out from the perspective of WEF as a whole, not from a single resource or two resources as many scholars currently consider. Therefore, this paper has special significance. In addition, there are many uncertain factors in the WEF system, the interval number multi-objective programming can accurately represent the uncertain information in the system. Therefore, the method proposed in this paper is innovative. However, there are some shortcomings in the research. Firstly, due to the numerous factors affecting the safety of the WEF system, changes in the objective function and constraint conditions will lead to changes in the optimization results. Therefore, how to determine a perfect objective function and select constraints, and build a more scientific and reasonable WEF system optimization model, needs to be further studied. Secondly, some parameters can only be converted with other indicators or preliminary predictions based on existing data due to the limitation of acquisition channels. The accuracy of the parameters needs to be improved.

## Figures and Tables

**Table 1:** Optimization Model Target Expectation Level of WEF System Security in Ordos.

Target	Expectation Level Lower Limit	Expectation Level Upper Limit	Expectation Level Tolerance
WEF system coordination deviation	0.02	0.179	0.159
Ecological environment goal	37319.66	37319.66	0

**Table 2:** Ordos WEF system security satisfaction and decision variable value.

Decision Variables	Lower Limit after Optimization	Upper Limit after Optimization
Satisfaction	0.884	0.915
Coal production (10,000 tons of standard coal)	46,489.41	46,429.5
Natural gas production (10,000 tons of standard coal)	3909.784	3300
Thermal power generation (10,000 tons of standard coal)	1267.48	1267.48
Hydropower power generation (10,000 tons of standard coal)	23.086	23.086
Wind power generation (10,000 tons of standard coal)	9.434	9.434
Food production (10,000 tons)	174.216	161.501
Utilization of water resources in coal production (10,000 tons)	14,347.20	14,347.20
Utilization of water resources in natural gas production (10,000 tons)	763.359	763.359
Utilization of water resources for thermal power generation (10,000 tons)	13,351.13	13,351.13
Utilization of water resources for hydroelectric power generation (10,000 tons)	996.16	996.16
Utilization of water resources in food production (10,000 tons)	110,862.1	110,862.1

## Supplementary Materials

The following are available online at https://www.mdpi.com/1660-4601/17/20/7508/s1. The specific values of social and economic parameters, water resource utilization parameters, energy production parameters, food production parameters and environmental pollution parameters are shown in Tables S1–S5.

## References

[1] Golam R. (2014). Food, water, and energy security in South Asia: A nexus perspective from the Hindu Kush Himalayan region. Environ. Sci. Policy.

[2] Putra F., Prajal P., Kropp J.P. (2020). A systematic analysis of Water-Energy-Food security nexus: A South Asian case study. Sci. Total Environ..

[3] Jia S.F. (2017). Dialogue with Researcher Jia Shaofeng-Seeking a win-win solution for water, energy and food security—A case study of Ordos City, Inner Mongolia Autonomous Region. China Water Resour..

[4] World Economic Forum (2011). Global Risks 2011 Report.

[5] Guillaume J.H.A., Kummu M., Eisner S., Varis O. (2015). Transferable Principles for Managing the Nexus: Lessons from Historical Global Water Modeling of Central Asia. Water.

[6] Yan X.X., Jiang D., Fu J.Y., Hao M. (2018). Assessment of sweet sorghum-based ethanol potential in China within the water–energy–food nexus framework. Sustainability.

[7] Chen J.F., Ding T.H., Li M., Wang H.M. (2020). Multi-objective optimization of a regional water–energy–food system considering environmental constraints: A case study of Inner Mongolia, China. Int. J. Environ. Res. Public Health.

[8] Endo A., Tsurita I., Burnett K., Orencio P.M. (2017). A review of the current state of research on the water, energy, and food nexus. J. Hydrol. Reg. Stud..

[9] Heard B.R., Miller S.A., Liang S., Xu M. (2017). Emerging challenges and opportunities for the food-energy-water nexus in urban systems. Curr. Opin. Chem. Eng..

[10] Chang Y., Xia P., Wang J.P. (2016). An overview of the water-energy-food relationship and its enlightenment to my country. Water Dev. Res..

[11] Amorim W.S.D., Valduga I.B., Ribeiro J.M.P., Williamson V.G., Krauser G.E., Magtoto M.K., Andrade J.B.S.O. (2018). The nexus between water, energy and food in the context of the global risks: An analysis of the interactions between food, water, and energy security. Environ. Impact Assess. Rev..

[12] Hoff H. Understanding the Nexus. Proceedings of the Background Paper for the Bonn 2011 Conference: The Water, Energy and Food Security Nexus.

[13] Nhamo L., Ndlela B., Nhemachena C., Mabhaudhi T., Mpandeli S., Matchaya G. (2018). The Water-Energy-Food Nexus: Climate Risks and Opportunities in Southern Africa. Water.

[14] Howells M., Hermann S., Welsch M., Bazilian M., Segerstrom R., Alfstad T., Gielen D., Rogner H., Fischer G., van Velthnizen H. (2013). Integrated analysis of climate change, land-use, energy and water strategies. Nat. Clim. Chang..

[15] Yan X.C., Fang L., Mu L. (2020). How does the water-energy-food nexus work in developing countries? An empirical study of China. Sci. Total Environ..

[16] Xu S.S., He W.J., Shen J.Q., Degefu D.M., Yuan L., Kong Y. (2019). Coupling and coordination degrees of the core water–energy–food nexus in China. Int. J. Environ. Res. Public Health.

[17] Chen J.F., Ding T.H., Wang H.M., Yu X.Y. (2019). Research on total factor productivity and influential factors of the regional water–energy–food nexus: A case study on Inner Mongolia, China. Int. J. Environ. Res. Public Health.

[18] Sahin O.Z., Stewart R.A., Richards R.G. Addressing the Water-Energy-Climate Nexus Conundrum: A System Approach. Proceedings of the 7th International Congress on Environment Modelling and Software.

[19] Li G.J., Li Y.L., Jia X.J., Du L., Huang D.H. (2016). Establishment and Simulation Study of System Dynamic Model on Sustainable Development of Water-Energy-Food Nexus in Beijing. Bus. Rev..

[20] Javadinejad S., Ostad-Ali-Askari K., Eslamian S. (2019). Application of Multi-Index Decision Analysis to Management Scenarios Considering Climate Change Prediction in the Zayandeh Rud River Basin. Water Conserv. Sci. Eng..

[21] Wang B., Li W., Huang G.H., Liu L., Ji L., Li Y. (2015). Urban water resources allocation under the uncertainties of water supply and demand: A case study of Urumqi, China. Environ. Earth Sci..

[22] Gong X.H., Zhang H.B., Ren C.F., Sun D.Y., Yang J.T. (2020). Optimization allocation of irrigation water resources based on crop water requirement under considering effective precipitation and uncertainty. Agric. Water Manag..

[23] Ju L.W., Tan Z.F., Li H.H., Tan Q.K., Yu X.B., Song X.H. (2016). Multi-objective operation optimization and evaluation model for CCHP and renewable energy based on hybrid energy system driven by distribute energy resources in China. Energy.

[24] Su M.R., Chen C., Yang Z.F. (2016). Urban energy structure optimization at the sector scale: Considering environmental impact based on life cycle assessment. J. Clean. Prod..

[25] Guo Z.L., Zhao X. (2017). Evolution and structure optimization of grain and cash crops in Shandong province based on 1986~2015 statistics data. J. China Agric. Resour. Reg. Plan..

[26] Cai Z., Qian W.R. (2017). Evaluation analysis and structural optimization of crop planting structure in northeast China. Fresenius Environ. Bull..

[27] Cao X., Shamxi A., Jin X.B., Zhou Y.K. (2011). Planting Structure Optimization in the Arid Area with Constrained Water Resources: A Case Study of Korla Xinjiang. Resour. Sci..

[28] Zhen J.L., Wu C.B., Liu X.R., Huang G.H., Liu Z.P. (2020). Energy-water nexus planning of regional electric power system within an inexact optimization model in Tangshan City, China. J. Clean. Prod..

[29] Chen C., Zheng X.T., Yu L., Huang G.H., Li Y.P. (2020). Planning energy-water nexus systems based on a dual risk aversion optimization method under multiple uncertainties. J. Clean. Prod..

[30] Zhang J., Campana P.E., Yao T., Zhang Y., Lundblad A., Melton F., Yan J.Y. (2017). The water-food-energy nexus optimization approach to combat agricultural drought: A case study in the United States. Appl. Energy.

[31] Karan E., Asadi S., Mohtar R., Baawain M. (2018). Towards the optimization of sustainable food-energy-water systems: A stochastic approach. J. Clean. Prod..

[32] Fan C.H., Lin C.Y., Hu M.C. (2019). Empirical framework for a relative sustainability evaluation of urbanization on the water–energy–food nexus using simultaneous equation analysis. Int. J. Environ. Res. Public Health.

[33] Li M., Fu Q., Singh V.P., Ji Y., Liu D., Li T.X. (2019). Stochastic multi-objective modeling for optimization of water-food-energy nexus of irrigated agriculture. Adv. Water Resour..

[34] Peng S.M., Zheng X.K., Wang Y., Jiang G.X. (2017). Collaborative optimization of water-energy-food in the Yellow River Basin. Adv. Water Sci..

[35] Xu B., Feng Z., Gates A.M. (2020). Multi-Objective Particle Swarm Optimization Algorithm for the Minimum Constraint Removal Problem. Int. J. Comput. Intell. Syst..

[36] Gao Y. (2010). Research on Some Problems of Multi-Objective Optimization. Master’s Thesis.

[37] Chen L. (2009). Research on Optimal Allocation of Logistics Resources in Pharmaceutical Enterprises. Master’s Thesis.

[38] Fan Y.L., Xia X.H. (2017). A multi-objective optimization model for energy efficiency building envelope retrofitting plan with. Appl. Energy.

[39] Ramirez-Atencia C., Camacho D. (2019). Constrained multi objective optimization for multi-UAV planning. J. Ambient Intell. Hum. Comput..

[40] Xu Y.D., Liu Y.M., Shen J.F. (2018). Multi-objective Track Maintenance Plan Model and Solution. J. Tongji Univ. Nat. Sci..

[41] Sun Y., Xu X.T. (2017). Evaluation of general land consolidation scheme based on grey multi objective decision model. J. Lanzhou Univ. Nat. Sci..

[42] Sharma U., Aggarwal S.S. (2018). Solving Fully Fuzzy Multi-objective Linear Programming Problem Using Nearest Interval Approximation. Int. J. Fuzzy Syst..

[43] Zhang Z.H., Tao J. (2015). Efficient micro immune optimization approach solving constrained nonlinear interval number programming. Appl. Intell..

[44] Jiang C., Han X., Li D. (2012). A New Interval Comparison Relation and Application in Interval Number Programming for Uncertain. CMC Comput. Mater. Continua.

[45] Yang L.B., Cui C.Y., Li Y.S. (2014). Analysis of water resources utilization status and potential in Ordos city. Yellow River.

[46] Peng S.M., He L.Y., Cui C.Y., Zheng X.K., Liu Z.S. (2014). Analysis and allocation pattern of water resources supply and demand in Ordos City. Yellow River.

[47] Kong D.W., Sun J. (2015). Interval Multi-Objective Evolutionary Optimization Theory and Application.

[48] Wang H.F., Wang M.L. (1997). A fuzzy multi objective linear programing. Fuzzy Sets Syst..

[49] Zhang X., Vesselinov V.V. (2017). Integrated modeling approach for optimal management of water, energy and food security nexus. Adv. Water Resour..

[50] OuYang X.Y. (2011). Research on the optimal development of energy structure in the western region—Based on the perspective of cost. Master’s Thesis.

[51] Li Y. (2016). Research on the production cost and benefit of main grain crops in Bayanzhuoer city. Master’s Thesis.

[52] Hong S.Y., Wang H.R., Lai W.L., Zhu Z.F. (2017). Spatial Analysis and Coordinated Development Decoupling Analysis of Energy-consumption Water in China. J. Nat. Resour..

[53] Li B., Zhang J.B. (2012). Research on the characteristics and spatial differences of carbon effect based on the change of agricultural land use in my country. Econ. Geogr..

[54] Tian Y., Li B., Zhang J.B. (2011). Research on stage characteristics and factor decomposition of carbon Emissions from agricultural land use in my country. J. China Univ. Geosci. Soc. Sci. Ed..

[55] Chen J.F., Chen L., Liu L.M., Zhi Y.L. Security Evaluation of Regional Water-Energy-Food System Based On Pressure-State-Response Model. Proceedings of the 15th Annual Conference of The Chinese Academy of Soft Science.

