# Supplementary material for: Optimization of Regional Water-Energy-Food Systems Based on Interval Number Multi-Objective Programming: A Case Study of Ordos, China
Source: Int J Environ Res Public Health. 2020 Oct 15;17(20):7508. doi: 10.3390/ijerph17207508 (PMC7602557; doi:10.3390/ijerph17207508)
Supplement: Supplementary file 1 [file ijerph-17-07508-s001.pdf]

Supplementary Materials:

The specific values of social and economic parameters, water resource utilization parameters, energy production parameters, food production parameters and environmental pollution parameters are shown in Table S1-S5.

Table S1. Social and Economic Parameters of WEF System Security in Ordos

Parameter Name	Parameter Value	Unit
Population	223	10,000 people
Cost coefficient of per unit of coal production	690	10,000yuan/ 10,000 tons of standard coal
Cost coefficient of per unit of natural gas production	860	10,000yuan /10,000 tons of standard coal
Cost coefficient per unit of thermal power generation	2440	10,000yuan /10,000 tons of standard coal
Cost coefficient per unit of hydraulic power generation	2030	10,000yuan /10,000 tons of standard coal
Cost coefficient per unit of wind power generation	4480	10,000yuan/10,000 tons of standard coal
Cost coefficient of unit food production	7.34×10^{-4}	10,000yuan /10,000 tons
Maximum cost of energy production	46500754.8	10,000yuan
Maximum cost of food production	0.12798	10,000yuan

Table S2. Safe Water Resource Utilization Parameters of WEF System in Ordos

Parameter Name	Parameter Value	Unit
Water resource utilization coefficient for coal mining	[0.1465,0.27]	10,000 tons/10,000 tons of standard coal
Water resource utilization coefficient for natural gas extraction	0.2034	10,000 tons/10,000 tons of standard coal
Water resource utilization coefficient for thermal power generation	[6.53,7.34]	10,000 tons/10,000 tons of standard coal
Water resource utilization coefficient for hydropower generation	[44.5972,65.58]	10,000 tons/10,000 tons of standard coal
Water resource utilization coefficient for food production	[561.8,720.09]	10,000 tons /10,000 tons
Maximum utilization of water resources in the city	167900	10,000 tons
Planning utilization of water resources in coal production	[7785.6,14348.88]	10,000 tons
Planning utilization of water resources for natural gas production	[630.948,762.88]	10,000 tons
Planning utilization of water resources for thermal power generation	[11874.34,13347.26]	10,000 tons
Planning utilization of hydropower water resources	[677.41,972.68]	10,000 tons
Planned utilization of water resources for food production	[97955.45,125554.8]	10,000 tons
Total planned utilization of water resources	151100	10,000 tons
Water demand for coal production	[6801.92,12535.97]	10,000 tons

Water demand for natural gas production	671.22	10,000 tons
Thermal power water demand	[7500,9053]	10,000 tons
Hydropower water demand	[356.78,996.16]	10,000 tons
Water demand for food production	[84270,108013.5]	10,000 tons

Table S3. Safe Energy Production Parameters of WEF System in Ordos

**Parameter Name**	**Parameter Value**	**Unit**
Planned coal production	[46429.5, 48572.4]	10,000 tons of standard coal
Planned natural gas production	[3300,5320]	10,000 tons of standard coal
Planned production of thermal power	1233.38	10,000 tons of standard coal
Hydropower planned production	15.19	10,000 tons of standard coal
Wind power planning production	9.44	10,000 tons of standard coal
Minimum coal production requirements	46429.5	10,000 tons of standard coal
Coal production capacity	57144	10,000 tons of standard coal
Minimum requirements for natural gas production	[3300,3990]	10,000 tons of standard coal
Minimum requirements for thermal power production	1200	10,000 tons of standard coal
Minimum wind power production requirements	8	10,000 tons of standard coal
Minimum power production requirements	2458	10,000 tons of standard coal
Proportion of clean energy	90%	%
Total energy consumption	4551.21	10,000 tons of standard coal
Energy self-sufficiency rate	85%	%

Table S4. Safe Food Production Parameters of WEF System in Ordos

Parameter Name	Parameter Value	Unit
Planned food production	174.36	10,000 tons
Food production per unit area of arable land	0.5874	10,000 tons / thousand hm^2
Cultivated land area per unit of food production	1.7	thousand hm^2/ 10,000 tons
Minimum requirements for food production	150	10,000 tons
Amount of fertilizer used per unit of food production	0.07	10,000 tons / 10,000 tons
Maximum value of fertilizer application	13	10,000 tons
Minimum per capita food production	0.4	10,000 tons /10,000person
Guaranteed food production area	224.64	thousand hm^2
Food consumption	130	10,000 tons
Food self-sufficiency rate	95%	%

Table S5. Security environmental pollution parameters of WEF system in Ordos

**Parameter Name**	**Parameter Value**	**Unit**
Coal carbon emission factor	0.7476	10,000 tons/10,000 tons of standard coal
Natural gas carbon emission factor	0.4435	10,000 tons/10,000 tons of standard coal
Thermal power generation carbon emission factor	0.287	10,000 tons/10,000 tons of standard coal
Hydropower carbon emission factor	0	10,000 tons/10,000 tons of standard coal
Wind power carbon emission factor	0	10,000 tons/10,000 tons of standard coal
Food production carbon emission factor	3.3	10,000 tons / 10,000 tons

Maximum carbon emissions from the energy subsystem	[57479,58127]	10,000 tons
Maximum carbon emissions from the food subsystem	575	10,000 tons
